# Acute Oral Toxicity of Tetrodotoxin in Mice: Determination of Lethal Dose 50 (LD50) and No Observed Adverse Effect Level (NOAEL)

**DOI:** 10.3390/toxins9030075

**Published:** 2017-02-24

**Authors:** Paula Abal, M. Carmen Louzao, Alvaro Antelo, Mercedes Alvarez, Eva Cagide, Natalia Vilariño, Mercedes R. Vieytes, Luis M. Botana

**Affiliations:** 1 Departamento de Farmacologia, Facultad de Veterinaria, Universidad de Santiago de Compostela, Lugo 27002, Spain; paula.abal@usc.es (P.A.); natalia.vilarino@usc.es (N.V.); 2 Laboratorio CIFGA S.A., Plaza Santo Domingo 20-5°, Lugo 27001, Spain; alvaro@cifga.es (A.A.); mercedes@cifga.es (M.A.); evacagide@cifga.es (E.C.); 3 Departamento de Fisiologia, Facultad de Veterinaria, Universidad de Santiago de Compostela, Lugo 27002, Spain; mmercedes.rodriguez@usc.es (M.R.V.)

**Keywords:** acute oral toxicity, European molluscs, lethal dose 50 (LD_50_), No Observed Adverse Effect Level (NOAEL), risk assessment, tetrodotoxin

## Abstract

Tetrodotoxin (TTX) is starting to appear in molluscs from the European waters and is a hazard to seafood consumers. This toxin blocks sodium channels resulting in neuromuscular paralysis and even death. As a part of the risk assessment process leading to a safe seafood level for TTX, oral toxicity data are required. In this study, a 4-level Up and Down Procedure was designed in order to determine for the first time the oral lethal dose 50 (LD_50_) and the No Observed Adverse Effect Level (NOAEL) in mice by using an accurate well-characterized TTX standard.

## 1. Introduction

Tetrodotoxin (TTX) and about 30 of its analogues are marine neurotoxins [1] first detected in puffer fishes [2], but recently identified in crustacea, gastropods, and bivalves [3]. TTX production is attributed to bacteria such as *Pseudomonas* and *Vibrio* spp. [4,5,6,7] that can reach animals through the food chain [8]. Bacteria may be eliminated in shellfish during cooking, but TTX is heat stable and cannot be destroyed in this process, and therefore is a risk to seafood consumers.

TTX has been responsible for human intoxications. It blocks sodium channels [9,10] and terminates nerve conduction and muscle action potentials [11], leading to progressive paralysis and, in extreme cases, to death from respiratory and heart failure [12,13].

Even though initially TTX distribution was limited to temperate climate zones, recent studies have determined its presence in European bivalves [14,15]. The presence of this toxin in shellfish creates a new problem due to the fact that there is no legal limit for TTX content in seafood within the European Union (EU). According to the current EU legislative requirements (Regulation 853/2004/EC; Regulation 854/2004/EC) only fishery products derived from poisonous fish of the family *Tetraodontidae* must not be placed on the market. The detection of TTX in shellfish and expansion of its geographical distribution require a risk assessment process leading to a safe seafood level for TTX as well as an adaptation of the legislation to control the presence of TTX in seafood. As a part of this process, information on the oral toxicological properties of TTX is needed.

## 2. Results

In the Up and Down Procedure, all mice administrated with 1000 µg/kg BW TTX by oral gavage died during the 2 h experiment. Therefore, 500 µg/kg BW was the dose for the second level, where four of the five mice died. In the third level, four of the seven mice treated with 250 µg/kg BW died, which lead to a reduction to 125 µg/kg BW for the fourth level, in which all of the mice survived (Table 1).

Apathy was a symptom quickly observed after the treatment with all doses. Piloerection, squint-eyes, or circling appeared less frequently. Over time, paralysis of extremities became more visible and close to death, some mice had seizures (Table 2). Mice treated with high doses died within minutes; they had seizures soon after the toxin administration that could mask prior paralysis of the extremities.

Death was usually by induced by cardiorespiratory failure. With the aim to determine the No Observed Adverse Effect Level (NOAEL), 9 mice were also administrated 75 and 25 µg/kg BW TTX. All those mice survived (Table 1) and showed no clinical signs of toxicity during the 2 h experiment.

**Figure 1:**
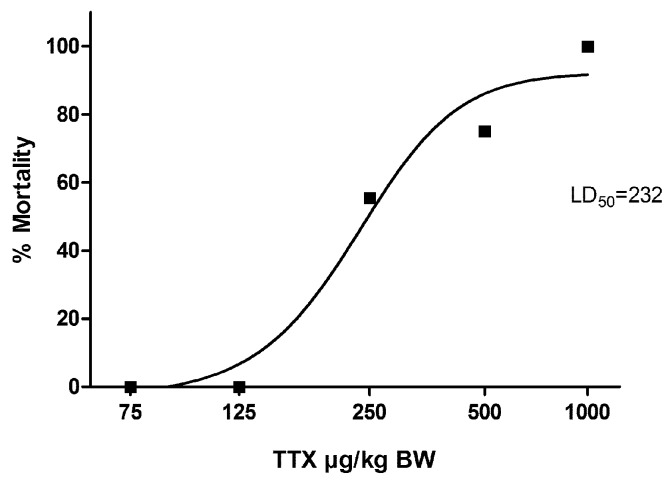
Dose response mortality curve of oral tetrodotoxin (TTX) in mice. Percentage lethality values are plotted against concentration of the toxin. The LD_50_ value is indicated.

The optimized 4-level Up and Down Procedure revealed a dose-dependent mortality induced by TTX. In Figure 1, all the results are presented in a semi-logarithmic scale as the percentage of mice mortality versus the administered toxin. Based on those data, the oral LD_50_ of TTX was estimated to be 232 µg/kg BW and the oral LD_100_ to be 1000 µg/kg BW by a nonlinear regression fitting procedure (GraphPad Prism 5., Version 5.01, ©1992-2007 GraphPad Software, Inc, La Jolla, CA, USA).

No macroscopic changes were observed in any organs of the mice treated with 25 µg/kg BW TTX, and only one mouse exposed to 75 µg/kg BW TTX had the stomach moderately dilated. However, all mice that received doses higher than 75 µg/kg BW had dilated stomachs with liquid and gas accumulation, even though the small intestine showed similar aspects to the controls. Only some mice administrated with 250 and 125 µg/kg BW TTX had duodenums that were moderately distended with liquid content. All mice treated with 1000 µg/kg TTX had cardiac stiffness, hence no blood was collected. TTX was successfully detected in blood samples from mice treated with 500 and 250 µg/kg BW (Table 3).

## 3. Discussion

TTX is a neurotoxin responsible for human poisoning due to the ingestion of food containing the toxin [16]. In fatal cases, humans show neurological signs within 2–3 h after exposure followed by death within 7–8 h due to respiratory failure and cardiovascular collapse [17]. Even though most intoxicated people should fully recover usually within 24 h [13], it was also reported that death may occur in less than 30 min [18], indicating a rapid rate of absorption from the gastrointestinal tract. Although hundreds of TTX intoxication cases have been published [19] it is difficult to find a correlation between toxin concentration and poisoning symptoms due to the scarce oral toxicity data. TTX is unequivocally toxic to mammals. In mice, the reported LD_50_ values were 2–10, 10–10.7, 12.5–16 µg/kg BW for intravenous, intraperitoneal i.p., and subcutaneous administration, respectively [13,14]. However, at present there are no oral toxicity data of purified TTX.

In this context and taking into account duration, rapidity of onset, and the severity of symptoms, the aim of the present study was to determine oral TTX toxicity in mice for 2 h by using a modified 4-level Up and Down Procedure. Our results indicated dose-dependent symptoms and mortality in mice exposed to TTX by gavage. Animals died of cardiorespiratory failure sometimes after a period of paralysis that supports the presence of biologically effective TTX concentrations in the blood [20]. In our study, the estimated oral LD_50_ = 232 µg/kg BW was 1.5–3 times lower than other values previously published [16,18,21]. It is important to note that this is the first report of *in vivo* studies using an accurate well-characterized TTX standard. This toxin at a dose of 75 and 25 µg/kg BW TTX did not cause any significant adverse effects, but higher doses induced at least clinical signs and macroscopic alterations at the gastrointestinal level. Therefore, according to these results we define for the first time the oral NOAEL of TTX for mice as 75 µg/kg BW.

## 4. Conclusions

TTX has now been identified in shellfish within the European waters [14,15], posing a problem to the seafood industry and a serious hazard to consumers. This fact should alert health authorities to the need for potential preventive measures. The oral LD_50_ (232 µg/kg BW) and NOAEL (75 µg/kg BW) values determined in this study may be used as a starting point for further risk assessment processes leading to a safe seafood level for TTX in the EU [22].

## 5. Materials and Methods

*In vivo* studies were performed with Swiss female mice weighing 18–21 g. All animal procedures described in the manuscript were carried out in conformity to European legislation (EU directive 2010/63/EU) and Spanish legislation (Real decreto 53/2013, Decreto 296/2008) and to the principles approved by the Institutional Animal Care Committee of the Universidad de Santiago de Compostela Code: AE-LU-002/13/FUN 01/TOX05/CON.AMB.[08]/LMBL8.

TTX used for all the experiments was obtained from CIFGA (Lugo, Spain). The stock solution was initially solved in aqueous [AcOH] = 1 × 10^−3^ M. Immediately before administration, TTX was diluted in 0.9% saline solution to achieve each dose (toxin carrier concentration <2.5%).

**Figure 2:**
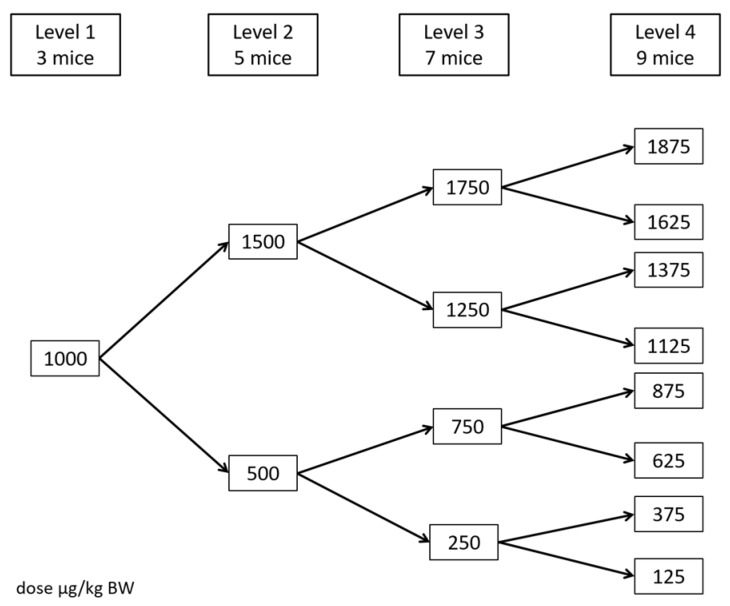
The 4-level Up and Down Procedure designed for the *in vivo* tetrodotoxin (TTX) study.

For the experimental procedure, mice were fasted overnight (12 h) with 5% glucosated serum *ad libitum*. The next morning they received a single oral dose of TTX by gavage (10 mL/kg) and were placed individually in metabolic cages for the following 2 h with free access to chow and water. A modified 4-level Up and Down Procedure [23] was set up to determine the lethal dose 50 (LD_50_) (Figure 2).

The number of mice was increased at each design level: 3 animals on the first level, 5 on the second, 7 on the third, and 9 on the fourth. The starting dose was 1000 µg/kg BW, and the dose for the next level was decreased if more than 50% of the mice died or increased if less than 50% of the mice died.

Symptoms were observed continuously during the whole experiment (2 h after toxin administration). Animals that survived were euthanized by CO_2_ inhalation. Macroscopic analysis was performed in all mice immediately after death. Time of death was indicated by last gasping breath. Blood was collected by cardiac puncture and stored at −80 °C until the time of analysis. Then, the extraction procedure was performed according to Tsujimura et al. 2015 [24].

The analysis of TTX was carried out using an Acquity UPLC BEH Amide 100 mm × 2.1 mm × 1.7 µm column (Waters, Milford, CT, USA), equipped with a 0.2 μm Acquity UPLC in-line filter. Mobile phases were as follows; A: water and B: acetonitrile (95% *v*/*v*) both containing 2 mM of ammonium formate and 3.6 mM of formic acid. The column temperature was set at 30 °C, flow was settled at 0.4 mL/min, and the injection volume was 5 µL. The chromatographic conditions consisted of initial conditions of 10%:90%, held for 1 min, then a linear gradient to 50%:50% over 3 min, held for 1 min. The column was then re-equilibrated using a linear gradient to 10%:90% over 0.1 min and then held for 1.5 min. Mass analysis was performed using a Xevo TQ MS mass spectrometer from Waters. (Manchester, UK) operated with the following parameters (ESI source): capillary potential 2.7 kV, cone voltage 39 V, desolvation temperature 350 °C, desolvation gas flow 850 L/h N_2_, cone gas flow 50 L/h N_2_, source temperature 150 °C, collision energy 40 V. Argon was used as the collision gas at 4.5 × 10^−3^ mbar. TTX was quantified against the certified reference standard CRM-03-TTXs sourced from Laboratorio CIFGA S.A. (Lugo, Spain. 81.2 ± 4.0 μmol TTXs/kg and 9.92 ± 0.70 μmol 4,9-anhTTX/kg, purity >96%) by using the following transitions: TTX and 4-epiTTX (*m*/*z* 320.2 > 178.1/162.0) and 4,9-anhydro TTX (302.1>162.0/150.0). Calibration standards were prepared from standard solution by dilution into acetonitrile/[AcOH] = 0.03 M (70:30 *v*/*v*) (standard dilution solvent) in polypropylene vials. A six point calibration was generated with variable concentrations ranging from 1 to 20 ng/mL.

## Figures and Tables

**Table 1:** Mortality induced by gavage administration of tetrodotoxin (TTX) to mice and survival times corresponding with each treatment.

Dose (µg/kg)	Mortality	Survival Times (min)
**1000**	3/3	7, 18, 37
**500**	4/5	58, 3, 2, 1
**250**	4/7	100, 7, 19, 54
**125**	0/9	>120
**75**	0/9	>120
**25**	0/9	>120

**Table 2:** Symptoms registered after tetrodotoxin (TTX) administration. Ratio between mice with the symptom versus the total mice treated.

Symptoms	TTX Dose (µg/kg)					
	1000	500	250	125	75	25
**Apathy**	3/3	5/5	7/7	9/9	0/9	0/9
**Piloerection**	0/3	0/5	1/7	2/9	0/9	0/9
**Paralysis of extremities**	3/3	2/5	2/7	0/9	0/9	0/9
**Seizures**	3/3	2/5	2/7	0/9	0/9	0/9
**Circling**	0/3	2/5	1/7	0/9	0/9	0/9
**Squint eyes**	0/3	1/5	0/7	0/9	0/9	0/9

**Table 3:** Tetrodotoxin (TTX) (ng/mL) quantified in blood samples by LC-MS/MS. Limit of detection (LOD) and limit of quantification (LOQ) of the method were 0.5 ng/mL and 1 ng/mL, respectively.

Dose (µg/kg)	TTX Level (ng/mL)
**500**	6.46 ± 1.91
**250**	1.29 ± 0.18
**125**	<LOQ
